# Supplementary material for: Population Genomics of Domesticated Cucurbita ficifolia Reveals a Recent Bottleneck and Low Gene Flow with Wild Relatives
Source: Plants (Basel). 2023 Nov 27;12(23):3989. doi: 10.3390/plants12233989 (PMC10707961; doi:10.3390/plants12233989)
Supplement: Supplementary file 1 [file plants-12-03989-s001.zip › plants-2585346-supplementary.pdf]

Electronic supplementary material of:

Population Genomics of Domesticated *Cucurbita ficifolia* Reveals a Recent Bottleneck and Low Gene Flow with Wild Relatives

Xitlali Aguirre-Dugua, Josué Barrera-Redondo, Jaime Gasca-Pineda, Alejandra Vázquez-Lobo, Andrea López-Camacho, Guillermo Sánchez-de la Vega, Gabriela Castellanos-Morales, Enrique Scheinvar, Erika Aguirre-Planter, Rafael Lira-Saade and Luis E. Eguiarte

Contact: xitlali.aguirre@conahcyt.mx, rlira@unam.mx, fruns@unam.mx

This file includes:

Supplementary Methodological Information

- Sequencing and assembly of *Cucurbita ficifolia* genome.
- Coalescent modelling parameters for *C. ficifolia* historical demography with FastSimCoal v2.6.
- Coalescent modelling parameters for assessing genealogical relationships among wild taxa, and gene flow between *C. ficifolia* and wild taxa with FastSimCoal v2.6.
- *Admixture* v1.3.0 analysis of the five-taxa dataset with 6 292 SNPs.

Supplementary Tables

Table S1. Assembly metrics of the genome of *Cucurbita ficifolia*.
Table S2. GenBank accession numbers of non-coding cpDNA sequences of *Cucurbita* taxa.
Table S3. Model selection of four hypothetical topologies relating wild xerophytic *C. x scabridifolia* to *C. foetidissima* and *C. pedatifolia*.
Table S4. Raw reads of Mexican *C. ficifolia* for whole genome sequencing available at BioProject PRJNA485527.
Table S5. Raw reads of GBS samples of the five taxa and outgroup included in this study.

Supplementary Figures

Figure S1. Models depicting different genealogical relationships among members of the xerophytic clade and gene flow among *C. ficifolia* and each of its wild relatives.
Figure S2. Assignation analysis of *C. ficifolia* with *Admixture* v1.3.0, based on 2 524 unlinked nuclear SNPs.
Figure S3. Distribution of the likelihood and the AIC of demographic models no. 2 and 3 of *C. ficifolia*.

Figure S4. Maximum Likelihood tree of *Cucurbita,* inferred from three non-coding chloroplast regions (*rpl20-rps12*, *petA-psbJ*, and *atpH-atpI* intergenic spacers).

Figure S5. Mismatch distribution analysis of *Cucurbita ficifolia* concatenated 200 SNP variants from plastome non-coding regions.

Figure S6. Cross-validation error for each of the $K = 2$ to $K = 15$ gene clusters of the five-taxa dataset with 6 292 unlinked nuclear SNPs.

Figure S7. Assignment of samples $K = 3$ to $K = 6$ gene pools of the five-taxa dataset with 6 292 unlinked nuclear SNPs.

Figure S8. Distribution of the likelihood and the AIC of the expected SFS under the best parameters of each of three genealogical relationships among *C. foetidissima*, *C. pedatifolia* and *C.* x *scabridifolia.*

Figure S9. Maximum likelihood tree of five *Cucurbita* taxa built from the concatenated 440 SNP plastome variants.

Figure S10. Leaf morphological variability in samples of *C. pedatifolia*, *C.* x *scabridifolia* and *C. foetidissima*.

Supplementary Data

- Commands used for the genome assembly of *Cucurbita ficifolia.*
- Code for SNP calling.
    - Nuclear data
    - Plastome data
- Code for FastSimCoal v2.6 demographic modelling.

Sequencing and assembly of *Cucurbita ficifolia* genome

Total DNA was obtained from leaves of a seed grown from a *C. ficifolia* fruit collected in Morelos (Mexico), and sequenced at the Vincent J. Coates Genomics Sequencing Laboratory in UC Berkeley (NIH S10 Instrumentation Grants S10RR029668 and S10RR027303) using an Illumina HiSeq4000 system with 500 bp and 1000 bp libraries, obtaining 11.8 Gb and 9.9 Gb of paired-end data, respectively.

In addition, we sequenced this sample at the University of Washington PacBio Sequencing Services, using three PacBio Sequel SMRT cells with a 20kb size-selected library, obtaining 23.4 Gb of sequencing data.

We performed quality filters to the Illumina sequences using the qualityControl.py script (https://github.com/Czh3/NGSTools), retaining the reads with a PHRED quality ≥ 30 in 85% of the sequence and an average PHRED quality ≥ 25. Illumina adapters were removed using SeqPrep (https://github.com/jstjohn/SeqPrep) and paired reads with an overlap ≥ 20 bp were merged.

The chloroplast and mitochondrial genomes were assembled with NOVOplasty [1], using the chloroplast genome of *C. argyrosperma* (GenBank CM014103.1) and the mitochondrial genome of *C. pepo* (GenBank NC_014050.1) as sequence seeds for the initial steps of the assembly [2,3]. We separated nuclear from organellar reads by mapping the Illumina and PacBio reads against the newly assembled organelle genomes using Hisat2 [4] and minimap2 [5]. Illumina nuclear reads were assembled into contigs using Platanus [6], which were then assembled into larger contigs using the PacBio Sequel reads and DBG2OLC [7]. We polished the DBG2OLC assembly by performing two iterations of minimap2 and racon [5] to map the PacBio reads and the Platanus contigs against the DBG2OLC contigs. We performed three additional polishing steps using PILON [8] and BWA mem [9] to map the Illumina reads against the genome assembly.

We performed a reference-guided scaffolding step using RaGOO [10] against the genome assembly of *C. maxima* [11]. We used PacBio corrected reads generated with CANU [12] to detect and correct contig mis-assemblies during the scaffolding step with RaGOO. We used a padding length of 100 bp to separate the contigs within the scaffolds. The

chromosome numbers in the final assembly were assigned in correspondence to the genome assembly of *C. moschata* [11].

The resulting *C. ficifolia* genome combined Illumina HiSeq4000 (90x coverage) and PacBio Sequel (97x coverage) reads for a final assembly in 640 contigs with an N50 contig size of 2.67 Mbp and a L50 of 27 contigs (Suppl. Table S1). We were able to anchor 97.4% of the genome assembly into 20 scaffolds corresponding to each of the chromosomes of *C. ficifolia*.

We performed a BUSCO analysis [13] against the *embryophyte odb9* database, detecting 93% of complete BUSCOs, 1.6% of fragmented BUSCOs and 5.4% of missing BUSCOs. This indicates that the quality of the genome assembly is comparable to other published *Cucurbita* genomes [2,11,14].

All the raw sequencing data and genome assembly of *C. ficifolia* are available in the National Center of Biotechnology Information under BioProject accession PRJNA485527.

Table S1. Assembly metrics of the genome of *Cucurbita ficifolia.*

Metrics	***Cucurbita ficifolia*** **genome assembly**
Total assembly size (bp)	240,667,112
No. of contigs	640
No. of scaffolds	70
Longest contig (bp)	10,261,322
Longest scaffold (bp)	20,756,987
N50 contig length (bp)	2,670,869
N50 scaffold length (bp)	11,298,682
L50 contig count	27 contigs
L50 scaffold count	9 scaffolds
No. of contigs > 1 kb	639 (99.8%)
No. of contigs > 10 kb	636 (99.4%)
No. of contigs > 100 kb	210 (32.8%)
No. of scaffolds > 10 kb	68 (97.1%)
No. of scaffolds > 100 kb	37 (52.9%)
No. of scaffolds > 1 Mb	20 (28.6%)
CG content	36.42%
Illumina read coverage	90x
PacBio read coverage	97x
Complete BUSCOs	93%
Fragmented BUSCOs	1.6%
Missing BUSCOs	5.4%

Coalescent modelling parameters for *C. ficifolia* historical demography with FastSimCoal v2.6

Parameter estimation was performed with initial uniform prior distribution values of all effective population sizes (*Ncurr, Nanc1, Nanc2*) ranging from N_e = 10 to 100 000, time to demographic change from 10 to 100 000 generations (without upper bound for the search space) and a mutation rate $\mu = 9.4 \times 10^{-7}$ per site per year.

Mutation rate was based on the formula $\mu = Ks/2T$ [15], where Ks = 2.476 is the number of synonymous substitutions in nuclear genes within eudicots [16] and T = 131.7 million years for the crown age of this plant group [17]. Considering that the SFS was built without invariant sites and that each SNP came from a 100 bp sequence, this value was corrected two orders of magnitude to obtain a final mutation rate of 9.4×10^{-7} per site per year.

For each model, we chose the run that showed the highest likelihood among 50 independent replicates. Once the best model was selected according to AIC values and likelihood distributions, we performed a parametric bootstrap for estimating a 95% confidence interval for our parameter values through the simulation of 100 SFS under the parameter values that maximized the likelihood of our data, and for each SFS we re-estimated the parameters with the highest likelihood among 20 independent runs.

Coalescent modelling parameters for assessing genealogical relationships among wild taxa, and gene flow between *C. ficifolia* and wild taxa

The issue of missing data was addressed by using the down-projection function in *dadi*, using the sample size that maximized the number of segregating sites [18] as evaluated with *easySFS* [19]. Down-sampled sizes were as follows: *C. foetidissima* 14 haploid samples, *C. pedatifolia* 30 haploid samples, *C. scabridifolia* 12 haploid samples, *C. radicans* 34 haploid samples and *C. ficifolia* 16 haploid samples.

Parameter estimation was performed with uniform prior distribution values of effective population sizes ranging from N_e = 10 to 100 000, number of generations to divergence from t = 10 to 100 000, and hybridization proportion from parental taxa of 0 to 1 (with a bounded upper limit). Migration (gene flow) rates were sampled from a loguniform distribution with values ranging from $1e^{-10}$ to $1e^{-1}$. It is worth mentioning that the upper limit in these ranges is used only for sampling the first random value of the parameter, for values can grow by 30% after each cycle, meaning that there is no upper limit for the parameter search space. We performed 50 replicate runs and chose the run with the highest likelihood.

The best model was selected according to the Akaike Information Criterion (AIC) , calculated as $AIC = 2k-2ln(likelihood)$, where k is the number of estimated parameters of the model and the *likelihood* is the probability of the data (the SFS) given the model [20]. We also compared the likelihood and AIC distributions of the two best models by running one million simulations of the SFS to get the likelihood distribution of the observed SFS under the best parameter values.

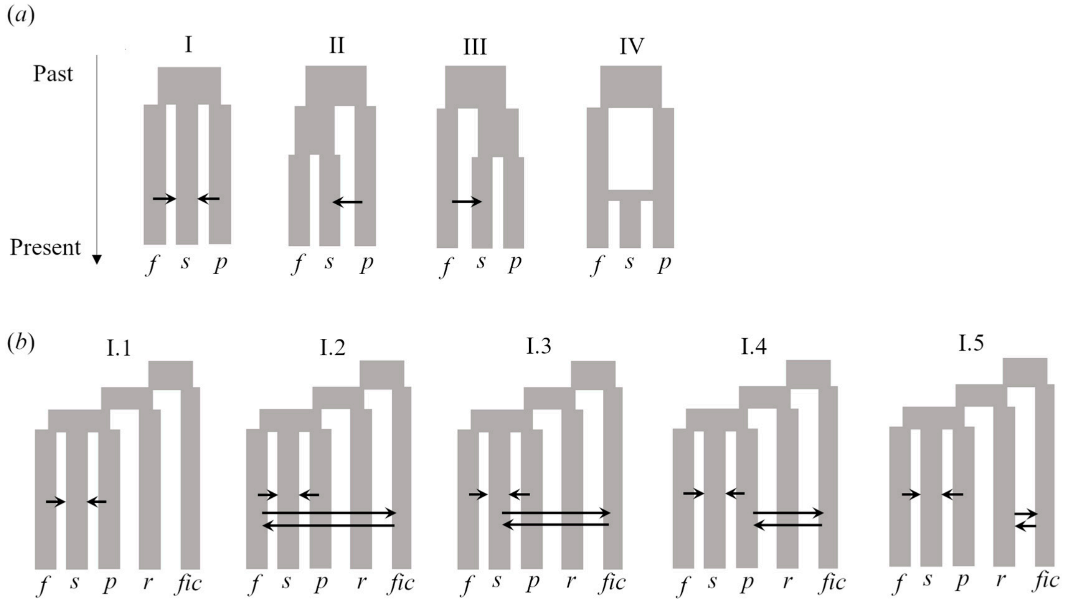

Figure S1. (*a*) First stage of model selection considering four possible genealogical relationships between *Cucurbita* x *scabridifolia* (*s*) and its putative parental species *C. foetidissima* (*f*) and *C. pedatifolia* (*p*), with arrows representing gene flow. Best fit model was model I. (*b*) Second stage of model selection with additional lineages of *C. radicans* (*r*) and *C. ficifolia* (*fic*), with possible gene flow between *C. ficifolia* and each of its wild relatives. The best fit model was model I.5 followed by model I.4 (Table 4).

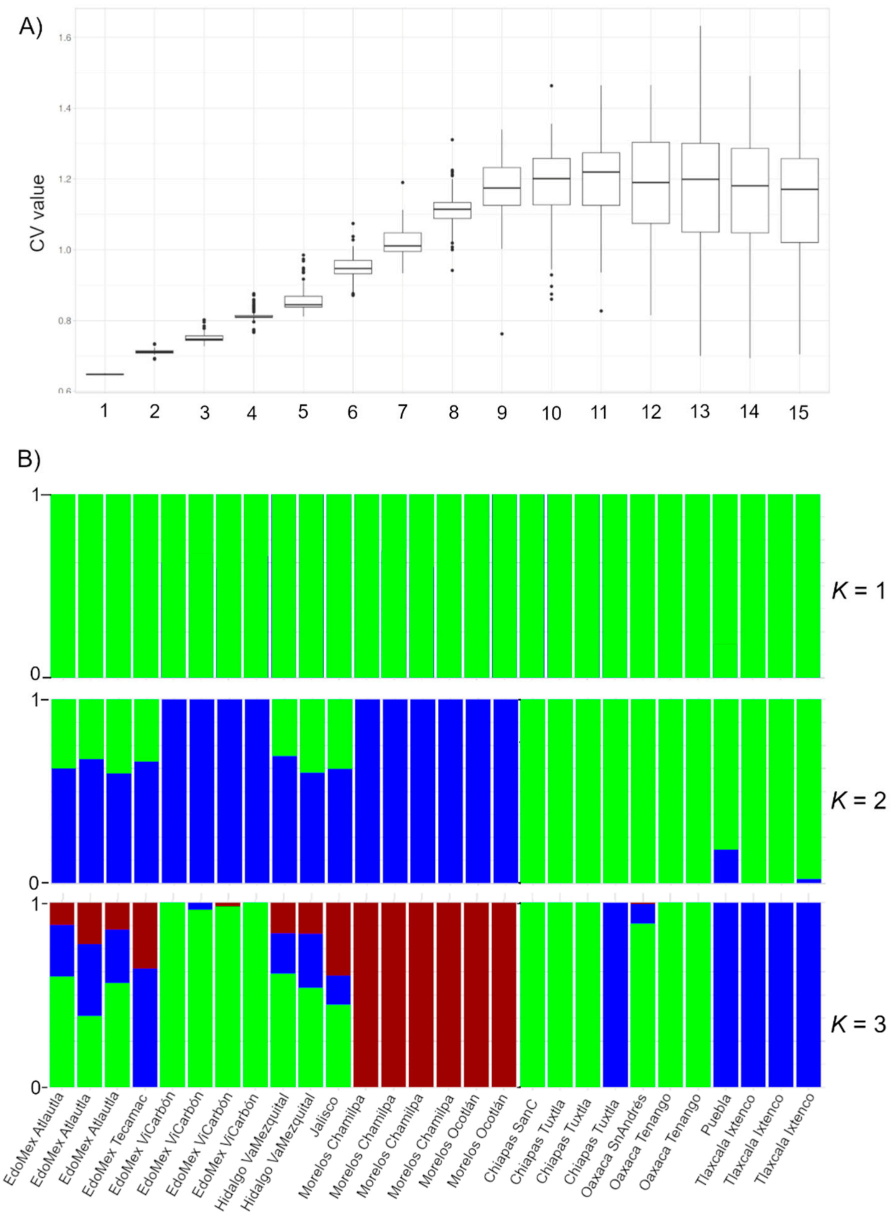

Figure S2. Assignment analysis of *C. ficifolia* with *Admixture* v1.3.0, based on 2 524 unlinked nuclear SNPs. (A) Cross-validation error for each of the *K* = 1 to *K* = 15 gene clusters. The lowest value of cross validation error (CV) points to the model that has the best predictive accuracy. (B) Assignation of individuals to *K* =1 to *K* = 3 clusters.

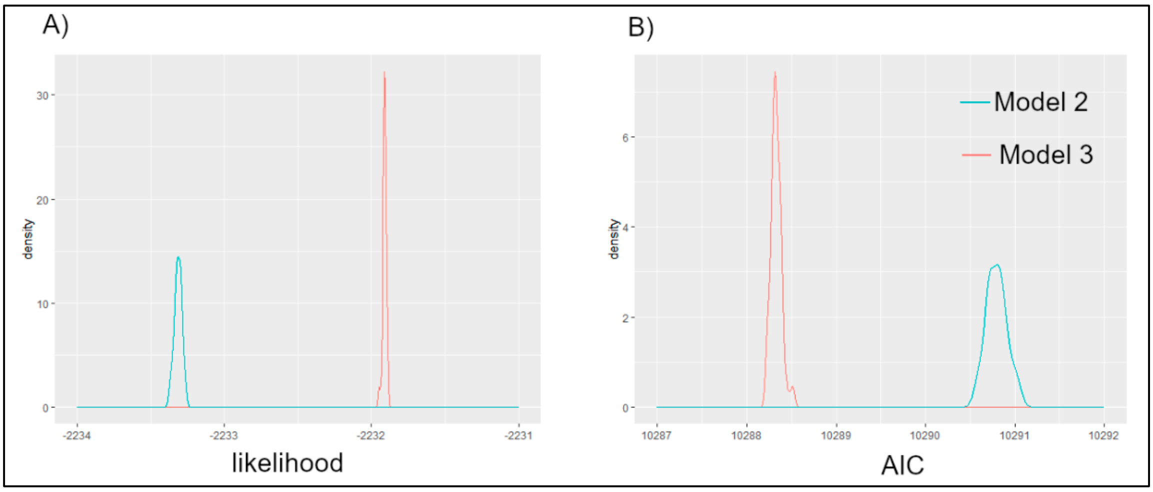

Figure S3. Distribution of (A) the likelihood and (B) the AIC of the expected SFS under the best parameters of model 2 (one population change with demographic contraction towards the present) and model 3 (two population changes with demographic growth followed by demographic contraction towards the present) in Mexican *C. ficifolia* obtained with 100 replicates of 1 million simulated SFS. Models are represented in Figure 3.

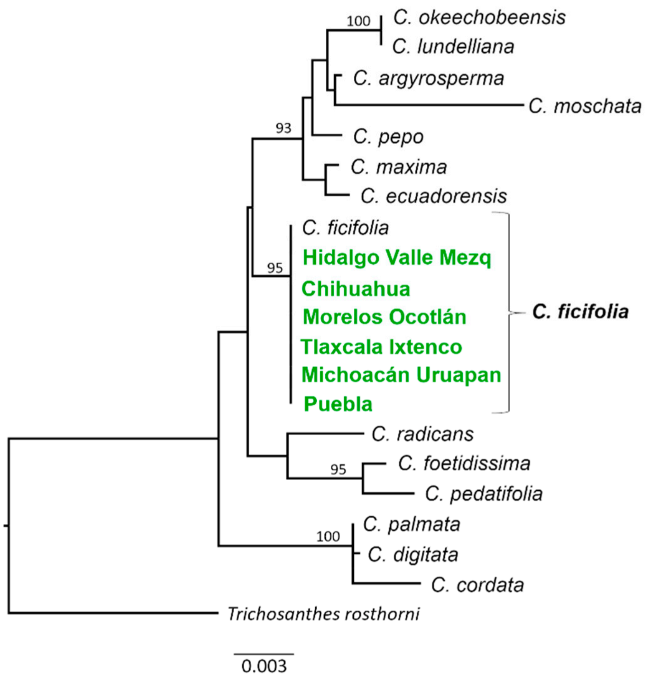

Figure S4. Maximum Likelihood tree of *Cucurbita,* inferred from three non-coding chloroplast regions (*rpl20-rps12*, *petA-psbJ*, and *atpH-atpI* intergenic spacers). Non-coding regions were amplified with primers from [21,22] at 50°-55°C of annealing temperature, sequenced at Macrogen (Korea), concatenated and aligned using MAFFT (https://mafft.cbrc.jp/alignment/software/). The Maximum Likelihood tree topology and support values (100 bootstrap replicates) were retrieved using with *PhyML* v3.0 (http://www.atgc-montpellier.fr/phyml/) with a GTR+G substitution model with 4 substitution rate classes, and a Gamma shape parameter estimated from the data. Only bootstrap supports >90 are shown. Green labels correspond to sequences of *C. ficifolia* generated for this work. Sequences of additional *Cucurbita* taxa were retrieved from GenBank (Table S2).

Table S2. GenBank accession numbers for non-coding cpDNA sequences of *Cucurbita* taxa. Samples of this study are highlighted in green colour.

**Taxon**	***rpl20-rps12***	***petA-psbJ***	***atpH-atpI***
Cficifolia_Puebla	OR270121	OR270132	OR270126
Cficifolia_MorelosOcotlan	OR270122	OR270133	OR270127
Cficifolia_MichoacanUruapan	--	OR270134	OR270128
Cficifolia_HidalgoValleMezquital	OR270123	OR270135	OR270129
Cficifolia_TlaxcalaIxtenco	OR270124	OR270136	OR270130
Cficifolia_Chihuahua	OR270125	OR270137	OR270131
C_ficifolia	MH470052.1	MH470035.1	OK336484.1
C_moschata	MH470042.1	MH470025.1	MH469991.1
C_palmata	MH470058.1	MH470041.1	MH470007.1
C_digitata	MH470057.1	MH470040.1	MH470006.1
C_cordata	MH470056.1	MH470039.1	MH470005.1
C_radicans	MH470055.1	MH470038.1	MH470004.1
C_foetidissima	MH470054.1	MH470037.1	MH470003.1
C_lundelliana	MH470049.1	MH470032.1	MH469998.1
C_argyrosperma	MH470044.1	MH470027.1	MH469993.1
C_ecuadorensis	MH470051.1	MH470034.1	MH470000.1
C_maxima	MH470050.1	MH470033.1	MH469999.1
C_pepo	MH470045.1	MH470028.1	MH469994.1
C_okeechobeensis	MH470048.1	MH470031.1	MH469997.1
C_pedatifolia	MH470053.1	MH470036.1	MH470002.1
Trichosanthes rosthorni	MT211650.1	MT211650.1	MT211650.1

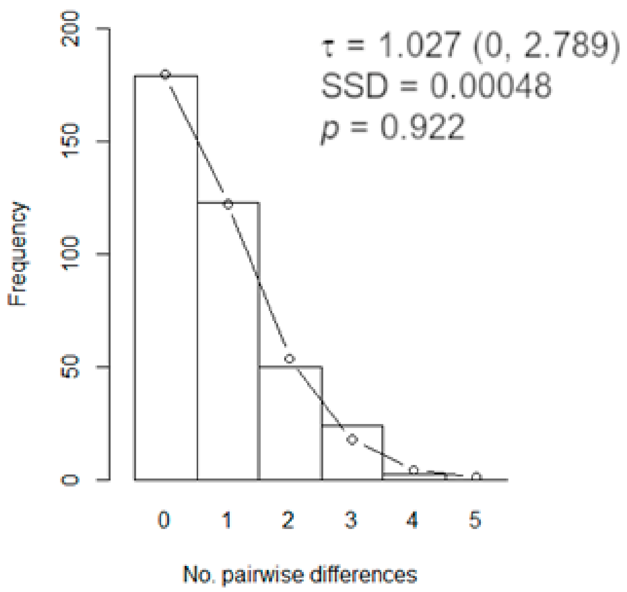

Figure S5. Mismatch distribution analysis of *Cucurbita ficifolia* plastome variants based on SNPs located in non-coding regions of the plastome (200 SNPs). The histogram represents the observed frequencies of pairwise differences among sequences, whereas the line represents the expected frequencies under a demographic expansion model. τ = tau parameter representing time to expansion (in mutational units), SSD = sum of squared deviations between observed and expected distributions. The expected distribution is modelled with 10 000 bootstrap replicates under a demographic expansion τ generations ago (95% CI is shown between parentheses).

Admixture v1.3.0 analysis of the five-taxa dataset with 6 292 SNPs

The *Admixture* analysis [23] recognized $K = 3$ nuclear gene pools as the best grouping in the dataset. The wild *Cucurbita radicans* samples were consistently clustered in their own pool (coloured red), whereas wild *C. foetidissima* and *C.* x *scabridifolia* samples were grouped in a second pool (blue) and the cultivated *C. ficifolia* and wild *C. pedatifolia* were grouped in a third pool (green). This pattern remained consistent when increasing the number of pools to $K = 4$, for *C. radicans* was divided in two pools while the other taxa kept their assignments to the same previously identified groups under $K = 3$. At $K = 5$ and $K = 6$, the analysis supported additional structure within *C. radicans* and *C. foetidissima*, and the differentiation of *C. x scabridifolia*.

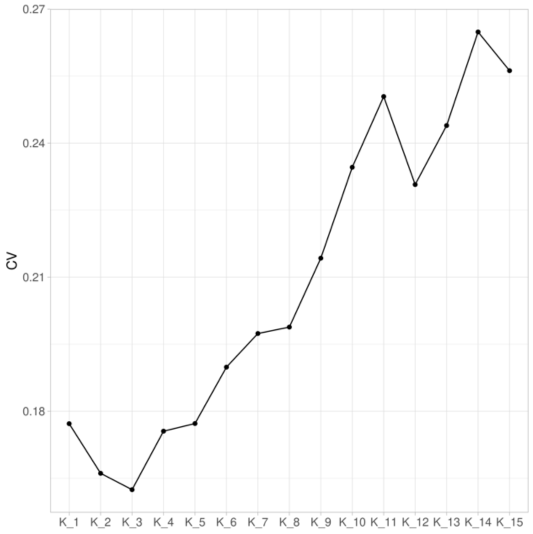

Figure S6. Cross-validation error for each of the $K = 2$ to $K = 15$ gene clusters of the five-taxa dataset, with 6 292 unlinked nuclear SNPs, as evaluated with *Admixture* v1.3.0. The lowest value of cross validation error (CV) points to the model that has the best predictive accuracy.

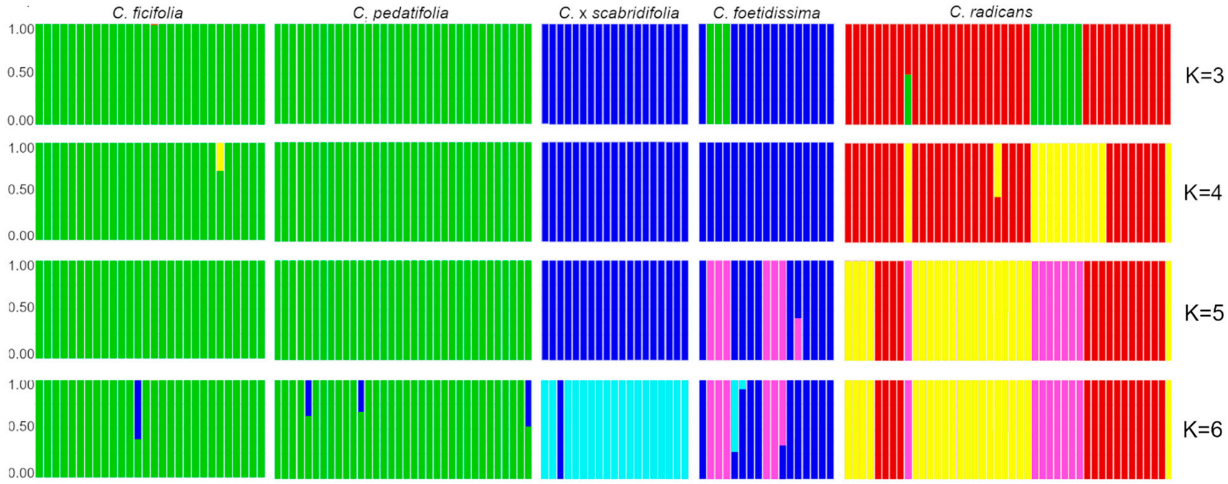

Figure S7. Assignment of samples $K = 3$ to $K = 6$ gene pools of the five-taxa dataset with 6 292 unlinked nuclear SNPs, as from *Admixture* v1.3.0. Vertical bars represent individuals, whereas each colour represents a gene pool.

Table S3. Model selection of four hypothetical topologies relating wild xerophytic *C.* x *scabridifolia* to *C. foetidissima* and *C. pedatifolia* (Figure S1). The three best models are shown in bold. LnMaxEstLhood = Maximum likelihood of the model, from the best run among 50 independent runs; Nparams = number of estimated parameters; AIC: Akaike Information Criterion; ΔAIC=Difference between the AIC of the model and the AIC of the best model among those evaluated.

Model	Genealogy	Gene flow to *C.* x *scabridifolia*	Ln MaxEstLhood	Nparams	AIC	ΔAIC	Rank
I	**Simultaneous divergence of three taxa from ancestor**	Absent	-15407.49	5	30824.98	398.11	7
		**Present**	**-15206.44**	**7**	**30426.87**	0	**1**
II	*C.* x *scabridifolia* sister to *C. foetidissima*	Absent	-15350.22	6	30712.44	285.57	5
		**Present**	**-15213.32**	**7**	**30440.64**	**13.77**	**2**
III	*C.* x *scabridifolia* sister to *C. pedatifolia*	Absent	-15403.96	6	30819.92	393.05	6
		Present	-15220.7	7	30455.4	28.53	4
IV	*C.* x *scabridifolia* hybrid of *C. foetidissima* and *C. pedatifolia*	**Absent**	**-15214.27**	**7**	**30442.54**	**15.67**	**3**

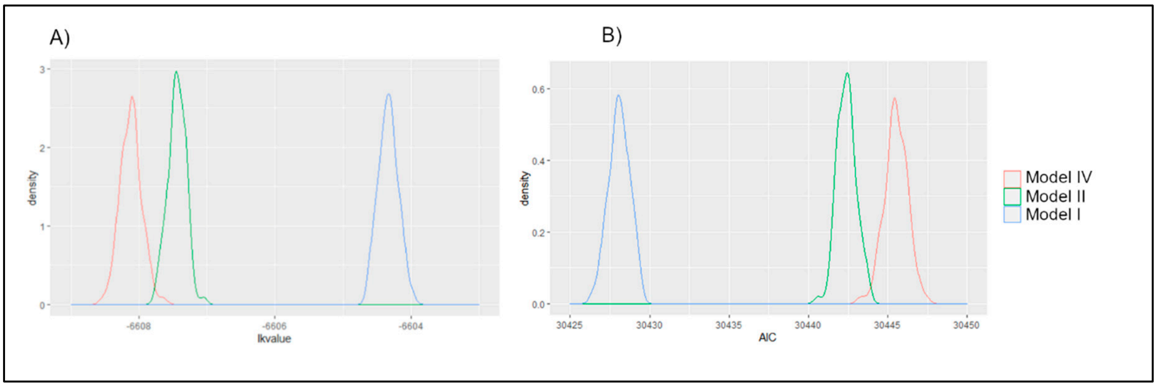

Figure S8. (A) Distribution of the likelihood and (B) of the AIC of the expected SFS under the best parameters of each of three genealogical relationships among *C. foetidissima*, *C. pedatifolia* and *C.* x *scabridifolia* obtained with 100 replicates of 1 million simulated SFS. Models are represented in Figure S1.

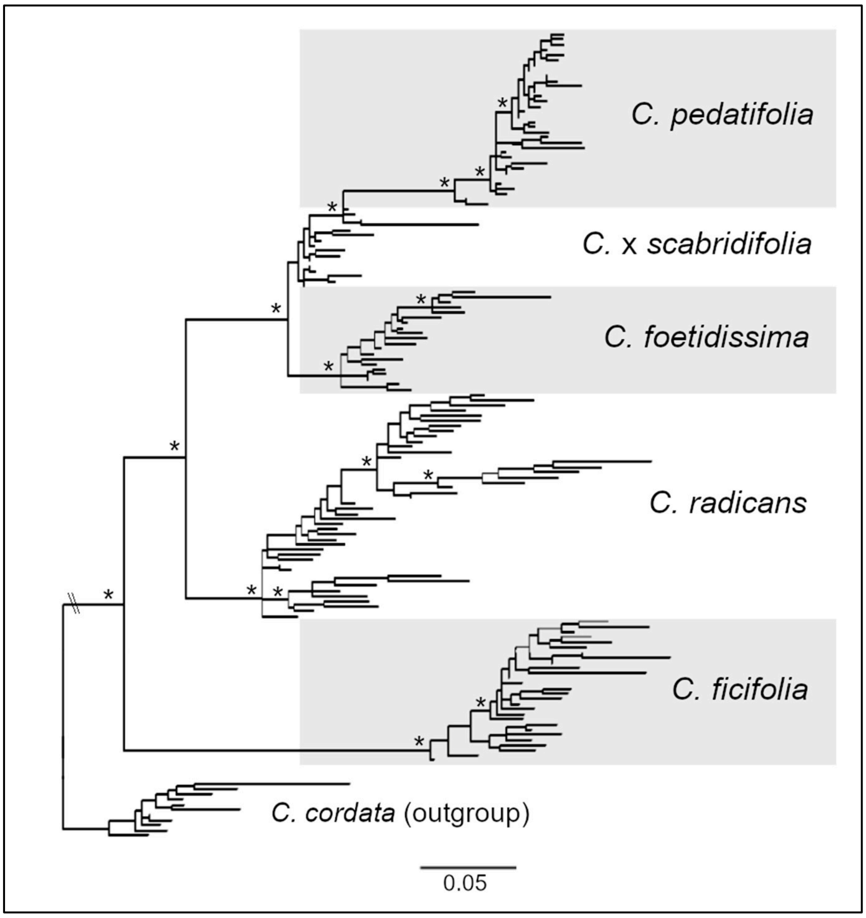

Figure S9. Maximum likelihood tree built from the concatenated 440 SNP plastome variants of samples. Nodes with bootstrap support >90 are shown with an asterisk.

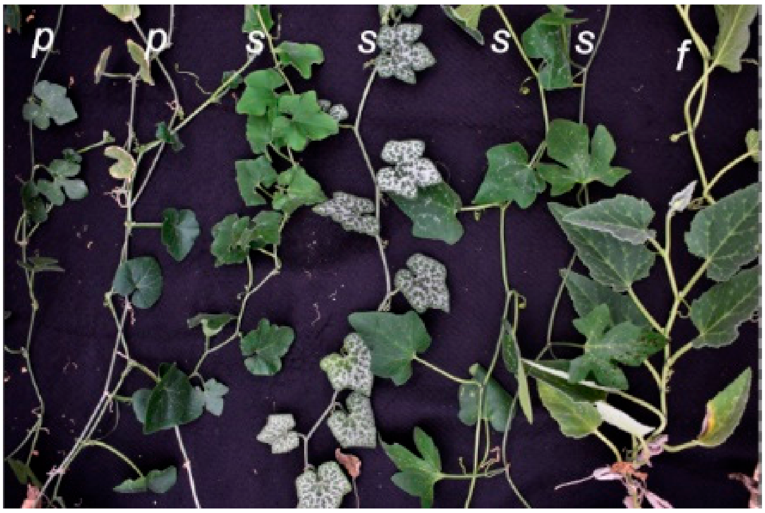

Figure S10. Leaf morphological variability in samples of *C. pedatifolia* (*p*), *C.* x *scabridifolia* (*s*) and *C. foetidissima* (*f*).

Table S4. Raw reads of Mexican *C. ficifolia* for whole genome sequencing available at BioProject PRJNA485527.

Individual ID	Location	Species	Latitude	Longitude	SRA accession
Cfic_WGS	Mexico: Cuernavaca, Morelos	*Cucurbita ficifolia*	18.9848 N	99.2370 W	SRR24660414
Cfic_WGS	Mexico: Cuernavaca, Morelos	*Cucurbita ficifolia*	18.9848 N	99.2370 W	SRR24660415
Cfic_WGS	Mexico: Cuernavaca, Morelos	*Cucurbita ficifolia*	18.9848 N	99.2370 W	SRR24660416

Table S5. Raw reads of GBS samples of the five taxa and outgroup included in this study.

Taxon	Bioproject	SRA accessions
Cucurbita ficifolia	PRJNA982063	SAMN35684881-SAMN35684916
Cucurbita pedatifolia	PRJNA982114	SAMN35685651-SAMN35685684
Cucurbita foetidissima	PRJNA982146	SAMN35686608-SAMN35686624
Cucurbita radicans	PRJNA976254	SAMN35361273-SAMN35361366
Cucurbita x scabridifolia	PRJNA485527	SAMN35686148-SAMN35686166
Cucurbita cordata	PRJNA982151	SAMN35686940-SAMN35686950

References

1. Dierckxsens N, Mardulyn P, Smits G. 2016 NOVOPlasty: *de novo* assembly of organelle genomes from whole genome data. *Nucleic Acids Res.* **45**, gkw955. (doi:10.1093/nar/gkw955)

2. Barrera-Redondo J, Ibarra-Laclette E, Vázquez-Lobo A, Gutiérrez-Guerrero YT, Sánchez de la Vega G, Piñero D, Montes-Hernández S, Lira-Saade R, Eguiarte LE. 2019 The genome of *Cucurbita argyrosperma* (silver-seed gourd) reveals faster rates of protein-coding gene and long noncoding RNA turnover and neofunctionalization within *Cucurbita*. *Mol. Plant* **12**, 506–520. (doi:10.1016/j.molp.2018.12.023)

3. Alverson AJ, Wei X, Rice DW, Stern DB, Barry K, Palmer JD. 2010 Insights into the evolution of mitochondrial genome size from complete sequences of Citrullus lanatus and *Cucurbita pepo* (Cucurbitaceae). *Mol. Biol. Evol.* **27**, 1436–1448. (doi:10.1093/molbev/msq029)

4. Kim D, Paggi JM, Park C, Bennett C, Salzberg SL. 2019 Graph-based genome alignment and genotyping with HISAT2 and HISAT-genotype. *Nat. Biotechnol.* **37**, 907–915. (doi:10.1038/s41587-019-0201-4)

5. Li H. 2018 Minimap2: pairwise alignment for nucleotide sequences. *Bioinformatics* **34**, 3094–3100. (doi:10.1093/bioinformatics/bty191)

6. Kajitani R *et al.* 2014 Efficient de novo assembly of highly heterozygous genomes from whole-genome shotgun short reads. *Genome Res.* **24**, 1384–1395. (doi:10.1101/gr.170720.113)

7. Ye C, Hill CM, Wu S, Ruan J, Ma Z (Sam). 2016 DBG2OLC: Efficient assembly of large genomes using long erroneous reads of the third generation sequencing technologies. *Sci. Rep.* **6**, 31900. (doi:10.1038/srep31900)

8. Walker BJ *et al.* 2014 Pilon: An integrated tool for comprehensive microbial variant detection and genome assembly improvement. *PLoS One* **9**, e112963. (doi:10.1371/journal.pone.0112963)

9. Li H, Durbin R. 2009 Fast and accurate short read alignment with Burrows-Wheeler transform. *Bioinformatics* **25**, 1754–60. (doi:10.1093/bioinformatics/btp324)

10. Alonge M, Soyk S, Ramakrishnan S, Wang X, Goodwin S, Sedlazeck FJ, Lippman ZB, Schatz MC. 2019 RaGOO: fast and accurate reference-guided scaffolding of draft genomes. *Genome Biol.* **20**, 224. (doi:10.1186/s13059-019-1829-6)

11. Sun H *et al.* 2017 Karyotype stability and unbiased fractionation in the paleo-allotetraploid *Cucurbita* genomes. *Mol. Plant* **10**, 1293–1306. (doi:10.1016/j.molp.2017.09.003)

12. Koren S, Walenz BP, Berlin K, Miller JR, Bergman NH, Phillippy AM. 2017 Canu: scalable and accurate long-read assembly via adaptive k -mer weighting and repeat separation. *Genome Res.* **27**, 722–736. (doi:10.1101/gr.215087.116)

13. Simão FA, Waterhouse RM, Ioannidis P, Kriventseva E V., Zdobnov EM. 2015 BUSCO: assessing genome assembly and annotation completeness with single-copy orthologs. *Bioinformatics* **31**, 3210–3212. (doi:10.1093/bioinformatics/btv351)

14. Montero-Pau J *et al.* 2018 De novo assembly of the zucchini genome reveals a whole-genome duplication associated with the origin of the *Cucurbita* genus. *Plant Biotechnol. J.* **16**, 1161–1171. (doi:10.1111/pbi.12860)

15. Wolfe KH, Li W-H, Sharp PM. 1987 Rates of nucleotide substitution vary greatly among plant mitochondrial, chloroplast, and nuclear DNAs. *Proc. Natl. Acad. Sci.* **84**, 9054–9058. (doi:10.1073/pnas.84.24.9054)

16. Drouin G, Daoud H, Xia J. 2008 Relative rates of synonymous substitutions in the mitochondrial, chloroplast and nuclear genomes of seed plants. *Mol. Phylogenet. Evol.* **49**, 827–831. (doi:10.1016/j.ympev.2008.09.009)

17. Magallón S, Gómez-Acevedo S, Sánchez-Reyes LL, Hernández-Hernández T. 2015 A metacalibrated time-tree documents the early rise of flowering plant phylogenetic diversity. *New Phytol.* **207**, 437–453. (doi:10.1111/nph.13264)

18. Gutenkunst RN, Hernandez RD, Williamson SH, Bustamante CD. 2009 Inferring the joint demographic history of multiple populations from multidimensional SNP frequency data. *PLoS Genet.* **5**. (doi:10.1371/journal.pgen.1000695)

19. Overcast I. 2020 easySFS.py.

20. Burnham KP, Anderson RP. 2004 Multimodel Inference: Understanding AIC and BIC in Model Selection. *Sociol. Methods Res.* **33**, 261–304. (doi:10.1177/0049124104268644)

21. Hamilton MB. 1999 Four primer pairs for the amplification of chloroplast intergenic regions with intraspecific variation. *Mol. Ecol.* **8**, 521–523.

22. Shaw J, Lickey EB, Schilling EE, Small RL. 2007 Comparison of whole chloroplast genome sequences to choose noncoding regions for phylogenetic studies in angiosperms: The tortoise and the hare III. *Am. J. Bot.* **94**, 275–288. (doi:10.3732/ajb.94.3.275)

23. Alexander DH, Novembre J, Lange K. 2009 Fast model-based estimation of ancestry in unrelated individuals. *Genome Res.* **19**, 1655–1664. (doi:10.1101/gr.094052.109)

Commands used for the genome assembly of *Cucurbita ficifolia*

```
### Remove low-quality reads from the Illumina data using qualityControl.py
(https://github.com/Czh3/NGSTools/blob/master/qualityControl.py)

python qualityControl.py -1 DPAVCF1_S16_L002_R1_001.fastq -2
DPAVCF1_S16_L002_R2_001.fastq -q 30 -p 85 -a 25 -o1
DPAVCF1_S16_L002_R1_001_FILTERED.fastq -o2
DPAVCF1_S16_L002_R2_001_FILTERED.fastq

python qualityControl.py -1 DPAVCF2_S17_L002_R1_001.fastq -2
DPAVCF2_S17_L002_R2_001.fastq -q 30 -p 85 -a 25 -o1
DPAVCF2_S17_L002_R1_001_FILTERED.fastq -o2
DPAVCF2_S17_L002_R2_001_FILTERED.fastq


### Remove adaptors and merge overlapping paired-end reads using SeqPrep
(https://github.com/jstjohn/SeqPrep)

SeqPrep -f DPAVCF1_S16_L002_R1_001_FILTERED.fastq -r
DPAVCF1_S16_L002_R2_001_FILTERED.fastq -1 DPAVCF1_R1_trimmed.fastq.gz -2
DPAVCF1_R2_trimmed.fastq.gz -3 discarded_DPAVCF1_R1.fastq.gz -4
discarded_DPAVCF1_R2.fastq.gz -s DPAVCF1_merged_reads.fastq.gz -o 20

SeqPrep -f DPAVCF2_S17_L002_R1_001_FILTERED.fastq -r
DPAVCF2_S17_L002_R2_001_FILTERED.fastq -1 DPAVCF2_R1_trimmed.fastq.gz -2
DPAVCF2_R2_trimmed.fastq.gz -3 discarded_DPAVCF2_R1.fastq.gz -4
discarded_DPAVCF2_R2.fastq.gz -s DPAVCF2_merged_reads.fastq.gz -o 20


### config file to assemble the chloroplast genome using NOVOPlasty. The
chloroplast genome of Cucurbita argyrosperma (NCBI accession CM014103) was used
as the seed sequence for the assembly.


Project:
-----------------------
Project name          = ficifolia
Type                  = chloro
Genome Range          = 120000-200000
K-mer                 = 39
Max memory            = 250
Extended log          = 0
Save assembled reads  = no
Seed Input            = CM014103.fasta
Reference sequence    = CM014103.fasta
Variance detection    = no
Heteroplasmy          =
HP exclude list       =
Chloroplast sequence  =

Dataset 1:
-----------------------
Read Length           = 151
```

```
Insert size           = 500
Platform              = illumina
Single/Paired         = PE
Combined reads        =
Forward reads         = DPAVCF1_R1_trimmed.fastq
Reverse reads         = DPAVCF1_R2_trimmed.fastq

Optional:
-----------------------
Insert size auto      = yes
Insert Range          = 1.8
Insert Range strict   = 1.3
```

Commands used to remove the organelle reads from the Illumina data using Hisat2. The mitochondrial genome f *Cucurbita pepo* (NCBI accession NC_014050) was used to remove the mitochondrial reads.

```
cat novoplasty/C.ficifolia_Chloroplast.fasta NC_014050.fasta > organelles.fasta

hisat2-build organelles.fasta organelles

hisat2 -x organelles -1 DPAVCF1_R1_trimmed.fastq -2 DPAVCF1_R2_trimmed.fastq -S DPAVCF1_trimmed_organelles.sam --un-conc DPAVCF1_trimmed_nucleus -p 8

hisat2 -x organelles -U DPAVCF1_merged_reads.fastq -S DPAVCF1_merged_organelles.sam --un DPAVCF1_merged_nucleus -p 8

hisat2 -x organelles -1 DPAVCF2_R1_trimmed.fastq -2 DPAVCF2_R2_trimmed.fastq -S DPAVCF2_trimmed_organelles.sam --un-conc DPAVCF2_trimmed_nucleus -p 8

hisat2 -x organelles -U DPAVCF2_merged_reads.fastq -S DPAVCF2_merged_organelles.sam --un DPAVCF2_merged_nucleus -p 8

mv DPAVCF1_trimmed_nucleus.1 DPAVCF1_trimmed_nucleus_R1.fastq

mv DPAVCF1_trimmed_nucleus.2 DPAVCF1_trimmed_nucleus_R2.fastq

mv DPAVCF2_trimmed_nucleus.1 DPAVCF2_trimmed_nucleus_R1.fastq

mv DPAVCF2_trimmed_nucleus.2 DPAVCF2_trimmed_nucleus_R2.fastq
```

```
### Command used to assemble the Illumina reads into contigs using Platanus


platanus assemble -t 20 -f DPAVCF1_merged_nucleus.fastq
DPAVCF1_trimmed_nucleus_R1.fastq DPAVCF1_trimmed_nucleus_R2.fastq
DPAVCF2_merged_nucleus.fastq DPAVCF2_trimmed_nucleus_R1.fastq
DPAVCF2_trimmed_nucleus_R2.fastq -o ficifolia -m 500
```

```
### Command used generate a hybrid assembly with the Platanus contigs and the
PacBio subreads using DBG2OLC


DBG2OLC k 17 KmerCovTh 5 MinOverlap 50 AdaptiveTh 0.01 RemoveChimera 1 Contigs
ficifolia_contig.fa f ficifolia_PacBio_subreads.fasta
```

```
### Polishing of DBG2OLC “backbone” assembly using minimap and racon


cat ficifolia_PacBio_subreads.fasta ficifolia_contig.fa > sequences.fasta


minimap2 -ax map-pb -t 10 backbone_raw.fasta sequences.fasta -o ficifolia1.sam


racon -u -t 10 sequences.fasta ficifolia1.sam backbone_raw.fasta >
consensus1.fasta


minimap2 -ax map-pb -t 10 consensus1.fasta sequences.fasta -o ficifolia2.sam


racon -u -t 10 sequences.fasta ficifolia2.sam consensus1.fasta >
consensus2.fasta
```

Loop used to run 3 iterations of PILON polishing by mapping the Illumina reads against the genome assembly

```
#!/bin/sh

ARCHIVO= consensus2.fasta
ITERACIONES=3
COUNTER=1

while [ ${ITERACIONES} -ge ${COUNTER} ]
	do
		bwa index ${ARCHIVO}
		bwa mem -t 20 ${ARCHIVO} DPAVCF1_trimmed_nucleus_R1.fastq DPAVCF1_trimmed_nucleus_R2.fastq > DPAVCF1_PE_trimmed_${COUNTER}.sam
		bwa mem -t 20 ${ARCHIVO} DPAVCF2_trimmed_nucleus_R1.fastq DPAVCF2_trimmed_nucleus_R2.fastq > DPAVCF2_PE_trimmed_${COUNTER}.sam
		bwa mem -t 20 ${ARCHIVO} DPAVCF1_merged_nucleus.fastq > DPAVCF1_merged_${COUNTER}.sam
		bwa mem -t 20 ${ARCHIVO} DPAVCF2_merged_nucleus.fastq > DPAVCF2_merged_${COUNTER}.sam
		samtools view -bS DPAVCF2_merged_${COUNTER}.sam > DPAVCF2_merged_${COUNTER}.bam
		samtools view -bS DPAVCF1_merged_${COUNTER}.sam > DPAVCF1_merged_${COUNTER}.bam
		samtools view -bS DPAVCF1_PE_trimmed_${COUNTER}.sam > DPAVCF1_PE_trimmed_${COUNTER}.bam
		samtools view -bS DPAVCF2_PE_trimmed_${COUNTER}.sam > DPAVCF2_PE_trimmed_${COUNTER}.bam
		samtools sort -o DPAVCF2_merged_${COUNTER}.sorted -@ 20 DPAVCF2_merged_${COUNTER}.bam
		samtools sort -o DPAVCF1_merged_${COUNTER}.sorted -@ 20 DPAVCF1_merged_${COUNTER}.bam
		samtools sort -o DPAVCF1_PE_trimmed_${COUNTER}.sorted -@ 20 DPAVCF1_PE_trimmed_${COUNTER}.bam
		samtools sort -o DPAVCF2_PE_trimmed_${COUNTER}.sorted -@ 20 DPAVCF2_PE_trimmed_${COUNTER}.bam
		samtools index DPAVCF1_merged_${COUNTER}.sorted
		samtools index DPAVCF1_PE_trimmed_${COUNTER}.sorted
```

```
samtools index DPAVCF2_merged_${COUNTER}.sorted

samtools index DPAVCF2_PE_trimmed_${COUNTER}.sorted

java -Xmx500000m -jar pilon-1.23.jar --genome ${ARCHIVO} --frags DPAVCF2_PE_trimmed_${COUNTER}.sorted --frags DPAVCF1_PE_trimmed_${COUNTER}.sorted --unpaired DPAVCF2_merged_${COUNTER}.sorted --unpaired DPAVCF1_merged_${COUNTER}.sorted --output iteracion_${COUNTER} --outdir salida_iteraciones --diploid --threads 20 > log_${COUNTER}_pilon

if [[ -f salida_iteraciones/iteracion_${COUNTER}.fasta ]]

    then

        rm DPAVCF2_merged_${COUNTER}.bam

        rm DPAVCF1_merged_${COUNTER}.bam

        rm DPAVCF1_PE_trimmed_${COUNTER}.bam

        rm DPAVCF2_PE_trimmed_${COUNTER}.bam

        rm DPAVCF1_merged_${COUNTER}.sorted

        rm DPAVCF1_PE_trimmed_${COUNTER}.sorted

        rm DPAVCF2_merged_${COUNTER}.sorted

        rm DPAVCF2_PE_trimmed_${COUNTER}.sorted

        rm DPAVCF1_merged_${COUNTER}.sorted.bai

        rm DPAVCF1_PE_trimmed_${COUNTER}.sorted.bai

        rm DPAVCF2_merged_${COUNTER}.sorted.bai

        rm DPAVCF2_PE_trimmed_${COUNTER}.sorted.bai

        rm DPAVCF2_merged_${COUNTER}.sam

        rm DPAVCF1_merged_${COUNTER}.sam

        rm DPAVCF1_PE_trimmed_${COUNTER}.sam

        rm DPAVCF2_PE_trimmed_${COUNTER}.sam

        rm ${ARCHIVO}.amb

        rm ${ARCHIVO}.ann

        rm ${ARCHIVO}.bwt

        rm ${ARCHIVO}.pac

        rm ${ARCHIVO}.sa

    else

        echo "ERROR: The file iteracion_${COUNTER}.fasta does not exist"

        exit 1

fi
```

```
            sed -i 's/_pilon//g' salida_iteraciones/iteracion_${COUNTER}.fasta
            ln -s salida_iteraciones/iteracion_${COUNTER}.fasta .
            ARCHIVO=iteracion_${COUNTER}.fasta
            let COUNTER+=1
      done

exit 0
```

Generate PacBio corrected reads using CANU

```
canu -d outfiles -p ficifolia genomeSize=250m -pacbio-raw
ficifolia_PacBio_subreads.fasta maxThreads=30 maxMemory=750 useGrid=false
```

Use the genome assembly of *Cucurbita maxima* (http://cucurbitgenomics.org/v2/organism/11) and the CANU-corrected reads to scaffold the PILON output into chromosomes

```
./build/scripts-3.7/ragoo.py -t 5 -R ficifolia.correctedReads.fasta.gz -T corr -
C iteracion_3.fasta Cmax_CHR.fa
```

Code and software files for SNP calling

Trimming and filtering Illumina 100 x SE reads

```
trimmomatic SE input.fastq.gz output_clean.fastq.gz
ILLUMINACLIP:Nextera_to_remove.fas:2:30:7 MAXINFO:80:0.2
```

Nuclear data

ipyrad params file for excluding reads mapping to organellar genomes

*Plastome and chondrome (cpDNA and mtDNA) reference sequences were concatenated in a single fasta file `organellarG.fasta`

*Nuclear reads are kept in resulting folder `organellar_REFexc_edits` under the name `SAMPLE-unmapped.fastq.gz`

* Note that the `[clust_threshold]` parameter is not relevant when `[assembly_method]` is `reference`

```
------- ipyrad params file (v.0.9.31)-------------------------------------------
organellar_REFexc            ## [0] [assembly_name]: Assembly name. Used to name output directories for assembly steps
home/clorop/ ## [1] [project_dir]: Project dir (made in curdir if not present)
                               ## [2] [raw_fastq_path]: Location of raw non-demultiplexed fastq files
                               ## [3] [barcodes_path]: Location of barcodes file
home/mydir/fastqfiles/*.fastq.gz      ## [4] [sorted_fastq_path]: Location of demultiplexed/sorted fastq files
reference                         ## [5] [assembly_method]: Assembly method (denovo, reference)
home/clorop/organellarG.fasta         ## [6] [reference_sequence]: Location of reference sequence file
ddrad                            ## [7] [datatype]: Datatype (see docs): rad, gbs, ddrad, etc.
GATC,RCAT                        ## [8] [restriction_overhang]: Restriction overhang (cut1,) or (cut1, cut2)
4                              ## [9] [max_low_qual_bases]: Max low quality base calls (Q<20) in a read
33                             ## [10] [phred_Qscore_offset]: phred Q score offset (33 is default and very standard)
6                              ## [11] [mindepth_statistical]: Min depth for statistical base calling
6                              ## [12] [mindepth_majrule]: Min depth for majority-rule base calling
10000                          ## [13] [maxdepth]: Max cluster depth within samples
0.85                           ## [14] [clust_threshold]: Clustering threshold for de novo assembly
0                              ## [15] [max_barcode_mismatch]: Max number of allowable mismatches in barcodes
0                              ## [16] [filter_adapters]: Filter for adapters/primers (1 or 2=stricter)
35                             ## [17] [filter_min_trim_len]: Min length of reads after adapter trim
2                              ## [18] [max_alleles_consens]: Max alleles per site in consensus sequences
0.05                           ## [19] [max_Ns_consens]: Max N's (uncalled bases) in consensus
0.05                           ## [20] [max_Hs_consens]: Max Hs (heterozygotes) in consensus
4                              ## [21] [min_samples_locus]: Min # samples per locus for output
0.2                            ## [22] [max_SNPs_locus]: Max # SNPs per locus
8                              ## [23] [max_Indels_locus]: Max # of indels per locus
0.5                            ## [24] [max_shared_Hs_locus]: Max # heterozygous sites per locus
0, 0, 0, 0                     ## [25] [trim_reads]: Trim raw read edges (R1>, <R1, R2>, <R2) (see docs)
0, 0, 0, 0                     ## [26] [trim_loci]: Trim locus edges (see docs) (R1>, <R1, R2>, <R2)
*                           ## [27] [output_formats]: Output formats (see docs)
                               ## [28] [pop_assign_file]: Path to population assignment file
                               ## [29] [reference_as_filter]: Reads mapped to this reference are removed in step 3
```

ipyrad params file for *C. ficifolia* SNP calling

```
------- ipyrad params file (v.0.9.31)-------------------------------------------
ficiref            ## [0] [assembly_name]: Assembly name. Used to name output directories for assembly steps
/home/mydir    ## [1] [project_dir]: Project dir (made in curdir if not present)
                               ## [2] [raw_fastq_path]: Location of raw non-demultiplexed fastq files
                               ## [3] [barcodes_path]: Location of barcodes file
/home/clorop/organellar_REFexc_edits/ficifolia/*-unmapped.fastq.gz    ## [4] [sorted_fastq_path]: Location of demultiplexed/sorted fastq files
reference                           ## [5] [assembly_method]: Assembly method (denovo, reference)
/home/mydir/Cficifolia_v1.chr.fa    ## [6] [reference_sequence]: Location of reference sequence file
ddrad                            ## [7] [datatype]: Datatype (see docs): rad, gbs, ddrad, etc.
GATC,RCAT                        ## [8] [restriction_overhang]: Restriction overhang (cut1,) or (cut1, cut2
4                              ## [9] [max_low_qual_bases]: Max low quality base calls (Q<20) in a read
33                             ## [10] [phred_Qscore_offset]: phred Q score offset (33 is default and very standard)
6                              ## [11] [mindepth_statistical]: Min depth for statistical base calling
6                              ## [12] [mindepth_majrule]: Min depth for majority-rule base calling
10000                          ## [13] [maxdepth]: Max cluster depth within samples
0.90                           ## [14] [clust_threshold]: Clustering threshold for de novo assembly
0                              ## [15] [max_barcode_mismatch]: Max number of allowable mismatches in barcodes
0                              ## [16] [filter_adapters]: Filter for adapters/primers (1 or 2=stricter)
35                             ## [17] [filter_min_trim_len]: Min length of reads after adapter trim
2                              ## [18] [max_alleles_consens]: Max alleles per site in consensus sequences
0.05                           ## [19] [max_Ns_consens]: Max N's (uncalled bases) in consensus
0.05                           ## [20] [max_Hs_consens]: Max Hs (heterozygotes) in consensus
4                              ## [21] [min_samples_locus]: Min # samples per locus for output
0.1                            ## [22] [max_SNPs_locus]: Max # SNPs per locus
8                              ## [23] [max_Indels_locus]: Max # of indels per locus
0.5                            ## [24] [max_shared_Hs_locus]: Max # heterozygous sites per locus
5,-5, 0, 0                     ## [25] [trim_reads]: Trim raw read edges (R1>, <R1, R2>, <R2) (see docs)
0, 0, 0, 0                     ## [26] [trim_loci]: Trim locus edges (see docs) (R1>, <R1, R2>, <R2)
*                           ## [27] [output_formats]: Output formats (see docs)
                               ## [28] [pop_assign_file]: Path to population assignment file
                               ## [29] [reference_as_filter]: Reads mapped to this reference are removed in step 3
```

ipyrad params file for five-taxa *(C. ficifolia, C. foetidissima, C. radicans, C. pedatifolia, C. x scabridifolia,* and outgroup *C. cordata)* nuclear SNP calling

```
------- ipyrad params file (v.0.9.31)-------------------------------------------
cucu_FIN            ## [0] [assembly_name]: Assembly name. Used to name output directories for assembly steps
home/xitlali/ficifolia/ipyrad_Final/ ## [1] [project_dir]: Project dir (made in curdir if not present)
                               ## [2] [raw_fastq_path]: Location of raw non-demultiplexed fastq files
                               ## [3] [barcodes_path]: Location of barcodes file
/home/clorop/organellar_REFexc_edits/alltaxa/*-unmapped.fastq.gz    ## [4] [sorted_fastq_path]: Location of demultiplexed/sorted fastq files
reference                         ## [5] [assembly_method]: Assembly method (denovo, reference)
/home/mydir/Cficifolia_v1.chr.fa  ## [6] [reference_sequence]: Location of reference sequence file
ddrad                            ## [7] [datatype]: Datatype (see docs): rad, gbs, ddrad, etc.
GATC,RCAT                        ## [8] [restriction_overhang]: Restriction overhang (cut1,) or (cut1, cut2)
4                              ## [9] [max_low_qual_bases]: Max low quality base calls (Q<20) in a read
33                             ## [10] [phred_Qscore_offset]: phred Q score offset (33 is default and very standard)
6                              ## [11] [mindepth_statistical]: Min depth for statistical base calling
6                              ## [12] [mindepth_majrule]: Min depth for majority-rule base calling
10000                          ## [13] [maxdepth]: Max cluster depth within samples
0.85                           ## [14] [clust_threshold]: Clustering threshold for de novo assembly
0                              ## [15] [max_barcode_mismatch]: Max number of allowable mismatches in barcodes
0                              ## [16] [filter_adapters]: Filter for adapters/primers (1 or 2=stricter)
35                             ## [17] [filter_min_trim_len]: Min length of reads after adapter trim
2                              ## [18] [max_alleles_consens]: Max alleles per site in consensus sequences
0.05                           ## [19] [max_Ns_consens]: Max N's (uncalled bases) in consensus
0.05                           ## [20] [max_Hs_consens]: Max Hs (heterozygotes) in consensus
4                              ## [21] [min_samples_locus]: Min # samples per locus for output
0.2                            ## [22] [max_SNPs_locus]: Max # SNPs per locus
8                              ## [23] [max_Indels_locus]: Max # of indels per locus
0.5                            ## [24] [max_shared_Hs_locus]: Max # heterozygous sites per locus
0, 0, 0, 0                     ## [25] [trim_reads]: Trim raw read edges (R1>, <R1, R2>, <R2) (see docs)
0, 0, 0, 0                     ## [26] [trim_loci]: Trim locus edges (see docs) (R1>, <R1, R2>, <R2)
*                           ## [27] [output_formats]: Output formats (see docs)
                               ## [28] [pop_assign_file]: Path to population assignment file
                               ## [29] [reference_as_filter]: Reads mapped to this reference are removed in step 3
```

Nuclear SNP filtering with *vcftools* and *plink*

```
vcftools --vcf ficiref.vcf --max-missing 0.7 --hwe 0.0001 --max-alleles 2 --recode --out ficiref_I

cat ficiref_I.recode.vcf | sed 's/RAD_//' > ficiref_I.vcf

#change to plink format
vcftools --vcf ficiref_I.vcf --plink

#add "Chr_" prefix to map file

cat out.map | sed 's/^/Chr_/' > out.map.2
mv out.map.2 out.map

#run plink for excluding sites with LD
plink --file out --indep-pairwise 100 100 0.25 --allow-extra-chr

#exclude sites within 250 bp distance
vcftools --vcf ficiref_I.vcf --exclude plink.prune.out --recode --out ficiref_II
vcftools --vcf ficiref_II.recode.vcf --thin 250 --recode --out ficiref_III
```

Population genetics statistics with *populations* module from *Stacks*

```
populations -V ficiref_III.recode.vcf -O ./ --min-maf 0.01 --fstats -k
```

Plastome data

ipyrad params file for five-taxa SNP calling on reference plastome (cpDNA)

```
------- ipyrad params file (v.0.9.31)-------------------------------------------
clorop_foet                       ## [0] [assembly_name]: Assembly name. Used to name output directories for assembly
steps
/home/clorop ## [1] [project_dir]: Project dir (made in curdir if not present)
                               ## [2] [raw_fastq_path]: Location of raw non-demultiplexed fastq files
                               ## [3] [barcodes_path]: Location of barcodes file
home/clorop/fastqfiles/*.fastq.gz     ## [4] [sorted_fastq_path]: Location of demultiplexed/sorted fastq files
reference                         ## [5] [assembly_method]: Assembly method (denovo, reference)
KT898810.fasta                              ## [6] [reference_sequence]: Location of reference sequence file
ddrad                            ## [7] [datatype]: Datatype (see docs): rad, gbs, ddrad, etc.
GATC,RCAT                         ## [8] [restriction_overhang]: Restriction overhang (cut1,) or (cut1, cut2)
5                              ## [9] [max_low_qual_bases]: Max low quality base calls (Q<20) in a read
33                             ## [10] [phred_Qscore_offset]: phred Q score offset (33 is default and very standard)
6                              ## [11] [mindepth_statistical]: Min depth for statistical base calling
6                              ## [12] [mindepth_majrule]: Min depth for majority-rule base calling
10000                          ## [13] [maxdepth]: Max cluster depth within samples
0.85                           ## [14] [clust_threshold]: Clustering threshold for de novo assembly
0                              ## [15] [max_barcode_mismatch]: Max number of allowable mismatches in barcodes
0                              ## [16] [filter_adapters]: Filter for adapters/primers (1 or 2=stricter)
35                             ## [17] [filter_min_trim_len]: Min length of reads after adapter trim
1                              ## [18] [max_alleles_consens]: Max alleles per site in consensus sequences
0.05                           ## [19] [max_Ns_consens]: Max N's (uncalled bases) in consensus
0.05                           ## [20] [max_Hs_consens]: Max Hs (heterozygotes) in consensus
4                              ## [21] [min_samples_locus]: Min # samples per locus for output
0.08                            ## [22] [max_SNPs_locus]: Max # SNPs per locus
8                              ## [23] [max_Indels_locus]: Max # of indels per locus
0.5                            ## [24] [max_shared_Hs_locus]: Max # heterozygous sites per locus
5, -5, 0, 0                     ## [25] [trim_reads]: Trim raw read edges (R1>, <R1, R2>, <R2) (see docs)
0, 0, 0, 0                     ## [26] [trim_loci]: Trim locus edges (see docs) (R1>, <R1, R2>, <R2)
v                        ## [27] [output_formats]: Output formats (see docs)
                               ## [28] [pop_assign_file]: Path to population assignment file
                               ## [29] [reference_as_filter]: Reads mapped to this reference are removed in step 3
```

Exclude SNPs falling in IR regions

```
#Positions of IR in C. foetidissima reference plastome KT898810
#IR1 94531-113016 eliminate
#SSC 113017-131011 keep
#IR2 131012-149497 eliminate
#LSC 149498-156776 keep

#In R:
IRmaskfoet<-c(rep(0,94530),rep(1,18486),rep(0,17995),rep(1,18486),rep(0,7279))
IRmaskfoet<-as.vector(IRmaskfoet)
write.table(rbind(IRmaskfoet),file="IRmaskfoet.txt",row.names=F,col.names=F,sep="")
#Add manually the header of fasta file ">KT898810" and save as *.fasta [fasta header must be same name as reference used
for creating *.vcf]
```

Filter plastome SNPs falling in IRs

```
vcftools --vcf clorop_foet.vcf --max-missing 0.8 --maxDP 800 --mask IRmaskfoet.txt --recode
```

Exclude SNPs falling in DNA-coding regions: follow the same procedure, using positions of CDS.

FastSimCoal demographic modelling

A) Generating SFS files

1) Run `easySFS` for choosing down-sampled size that maximizes number of segregating sites. See https://githubhot.com/index.php/repo/isaacovercast/easySFS

```
./easySFS.py -a -i mydata.vcf -p mydata_pops.txt –-preview
```

`Mydata_pops.txt` file follows the format (sample names should correspond to samples in vcf file, "`out`" represents outgroup):

```
Sample1       popA
Sample2       popA
Sample3       popB
Sample4       popB
Sample5       popC
Sample6       popC
Sample10      out
```

Convert vcf file to dadi input and create *.data file. See https://github.com/wk8910/bio_tools/blob/master/01.dadi_fsc/00.convertWithFSC/convert_vcf_to_dadi_input.pl

```
perl convert_vcf_to_dadi_input.pl mydata.vcf mydata_pops.txt
```

2) Use *.data file for creating SFS

```
#!/usr/bin/env python
import dadi

#UNFOLDED SFS
#Generate dictionary from *.data file
dd_mydata = dadi.Misc.make_data_dict('mydata.vcf.data')


#Create 2D SFSs
#For three demes, FastSimCoal requires the following SFSs: 1_0, 2_0, 2_1 (first index corresponds to rows, second index corresponds to columns)
#Define which deme corresponds to each index. For instance: 0=popA, 1=popB, 2=popC
#In "projections" include the down-sampled size previously identified
```

```
#If "polarized = True", you need in the data dictionary a group of samples labelled as "out". The resulting SFS will be unfolded. If "polarized = False", there are no samples representing an outgroup and the resulting SFS will be folded.
#Make sure to write the demes and their sample sizes in the right order to obtain the correct distribution of allele counts in rows/columns according to FastSimCoal indexes (1_0 vs. 0_1 for instance)

#fs_unfold_1_0 = dadi.Spectrum.from_data_dict(dd_mydata, ['popB','popA'], projections = [20,15], polarized = True)
#fs_unfold_2_0 = dadi.Spectrum.from_data_dict(dd_mydata, ['popC','popA'], projections = [30,15], polarized = True)
#fs_unfold_2_1 = dadi.Spectrum.from_data_dict(dd_mydata, ['popC','popB'], projections = [30,20], polarized = True)

#Create Multidimensional 3D spectrum. Index order should be 0_1_2
fs_unfold_0_1_2 = dadi.Spectrum.from_data_dict(dd_mydata, ['popA','popB','popC'], projections = [15,20,30], polarized = True)

#Export spectra to files
fs_unfold_1_0.to_file('mySFS_1_0.txt')
fs_unfold_0_1_2.to_file('mySFS_0_1_2.txt')
```

3) Edit manually the SFS to adjust to FastSimCoal format.

If you are dealing with 2D SFS:

- Remove last line of 1s and 0s.
- The first row indicates sample size of each deme + 1, in the format rows/columns. In our `mySFS_1_0.txt` example, sample size was 20 (popB) and 15 (popA). The SFS must indicate [21,16], and this is the size of the SFS matrix: 21 rows, 16 columns.
- Change data in one-line format to matrix format with corresponding number of rows and columns (in the example, that would be 21 x 16).
- Add row and column names as in FastSimCoal format. For rows: `d1_0 d1_1 (…) d1_20`, for columns: `d0_0 d0_1 (…) d0_15`
- First row must include the text "`1 observation`" (without quotes)
- Matrix begins in the second row.
- Make sure your end-lines are Linux.

If you are dealing with multidimensional SFS:

- Remove last line of 1s and 0s.
- First row must include the text "`1 observation`" (without quotes).
- Second row must indicate number of demes and sample size of each of them.
- Third row must include the SFS in a single, continuous line.
- Make sure your end-lines are Linux.

You have created the SFS *.obs files required by FastSimCoal. Please refer to FastSimCoal manual for more details on SFS file format.

B) FastSimCoal files for *Cucurbita ficifolia* historical demography

Run FastSimCoal with 50 independent replicates

```
#!/bin/bash

#One working directory with files *.obs *.est and *.tpl inside
#If using folded SFS, use -m option and *_MAFpop0.obs file
#If using unfolded SFS, use -d option and *_DAFpop0.obs file

PREFIX="MyModel"

 for i in {1..50}
 do
   mkdir run$i
```

```
   cp *.tpl *.est *.obs run$i
   cd run$i
   fsc26 -t ${PREFIX}.tpl -e ${PREFIX}.est -M -m -C 1 -n 500000 -L 70
   rm *.tpl *.est *.obs
   cd ..
 done
```

Constant population size model

*.est file

```
// Priors and rules file
// *********************

[PARAMETERS]
//#isInt? #name   #dist.#min  #max
//all Ns are in number of haploid individuals
1  NPOP  unif 10  100000 output

[RULES]

[COMPLEX PARAMETERS]
```

*.tpl file

```
//Number of population samples (demes)
1
//Population effective sizes (number of genes)
NPOP
//Sample sizes
18
//Growth rates: negative growth implies population expansion
0
//Number of migration matrices : 0 implies no migration between demes
0
//historical event: time, source, sink, migrants, new size, new growth rate, migr. matrix
1 historical event
300000 0 0 0 1 0 0
//Number of independent loci [chromosome]
1 0
//Per chromosome: Number of linkage blocks
1
//per Block: data type, num loci, rec. rate and mut rate + optional parameters
FREQ 1 0 9.4e-7 OUTEXP
```

One demographic change model

*.est file

```
// Priors and rules file
// *********************

[PARAMETERS]
//#isInt? #name   #dist.#min  #max
//all Ns are in number of haploid individuals
1  NPOP  unif 10  100000 output
1  NANC1  unif 10 100000  output
1  TCHAN  unif 10  100000 output


[RULES]

[COMPLEX PARAMETERS]
0  RESIZE  =  NANC1/NPOP  hide
```

*.tpl file

```
//Number of population samples (demes)
1
//Population effective sizes (number of genes)
NPOP
//Sample sizes
18
//Growth rates          : negative growth implies population expansion
0
```

```
//Number of migration matrices : 0 implies no migration between demes
0
//historical event: time, source, sink, migrants, new size, new growth rate, migr. matrix
1  historical event
TCHAN 0 0 0 RESIZE 0 0
//Number of independent loci [chromosome]
1 0
//Per chromosome: Number of linkage blocks
1
//per Block: data type, num loci, rec. rate and mut rate + optional parameters
FREQ 1 0 9.4e-7 OUTEXP
```

Two demographic changes model

*.est file

```
// Priors and rules file
// *********************

[PARAMETERS]
//#isInt? #name   #dist.#min  #max
//all Ns are in number of haploid individuals
1  NCUR   unif 10  100000 output
1  NANC2  unif 10  100000 output
1  NANC1  unif 10  100000 output
1  TCHAN  unif 10  100000 output
1  TEND$  unif 10  100000 hide

[RULES]

[COMPLEX PARAMETERS]
0 RESCHAN    = NANC1/NCUR hide
0 RESENDC = NANC2/NANC1 hide
1 TENDC = TCHAN+TEND$ output
```

*.tpl file

```
//Number of population samples (demes)
1
//Population effective sizes (number of genes)
NCUR
//Sample sizes
18
//Growth rates         : negative growth implies population expansion
0
//Number of migration matrices : 0 implies no migration between demes
0
//historical event: time, source, sink, migrants, new size, new growth rate, migr. matrix
2  historical event
TCHAN 0 0 0 RESCHAN 0 0
TENDC 0 0 0 RESENDC 0 0
//Number of independent loci [chromosome]
1 0
//Per chromosome: Number of linkage blocks
1
//per Block: data type, num loci, rec. rate and mut rate + optional parameters
FREQ 1 0 9.4e-7 OUTEXP
```

C) FastSimCoal parameter files for xerophytic taxa genealogical relationships

Note: the following models include gene flow from putative parental species to *C.* x *scabridifolia*. For the same models without gene flow, no migration matrix must be included in *.tpl file and no migration rate priors in *.est file.

```
//Number of migration matrices : 0 implies no migration between demes
0
```

Model I. Simultaneous divergence of three taxa

*. est file

```
// Priors and rules file
// *********************

[PARAMETERS]
//#isInt? #name   #dist.#min  #max
```

```
//all Ns are in number of haploid individuals
1  NPOPPED  unif 10  100000 output
1  NPOPSCA  unif 10  100000 output
1  NPOPFOE  unif 10  100000 output
1  NANC     unif 10  100000 output
1  TDIV     unif 10  100000 output
0  MIGSF logunif  1e-20 1e-1 output
0  MIGSP logunif  1e-20 1e-1 output

[RULES]

[COMPLEX PARAMETERS]
0    RESIZE  =  NANC/NPOPPED  hide
```

*.tpl file

```
//Number of population samples (demes)
3
//Population effective sizes (number of genes)
NPOPFOE
NPOPSCA
NPOPPED
//Sample sizes
14
12
30
//Growth rates          : negative growth implies population expansion
0
0
0
//Number of migration matrices : 0 implies no migration between demes
2
//Migration matrix 0
0 0 0
MIGSF 0 MIGSP
0 0 0
//Migration matrix 1: No migration
0 0 0
0 0 0
0 0 0
//historical event: time, source, sink, migrants, new size, new growth rate, migr. matrix
3  historical event
TDIV 0 2 1 1 0 1
TDIV 1 2 1 1 0 1
TDIV 2 2 1 RESIZE 0 1
//Number of independent loci [chromosome]
1 0
//Per chromosome: Number of linkage blocks
1
//per Block: data type, num loci, rec. rate and mut rate + optional parameters
FREQ 1 0 2.5e-8 OUTEXP
```

Model II. *C. x scabridifolia* as sister to *C. foetidissima*, receiving gene flow from *C. pedatifolia*

*.est file

```
// Priors and rules file
// *********************

[PARAMETERS]
//#isInt? #name   #dist.#min  #max
//all Ns are in number of haploid individuals
1  NPOPPED  unif 10  100000 output
1  NPOPSCA  unif 10  100000 output
1  NPOPFOE  unif 10  100000 output
1  NANC     unif 10  100000 output
1  TDIVSF   unif 10  100000 output
1  DELTA_T  unif 10  100000 hide
0  MIGSP logunif  1e-20 1e-1 output


[RULES]

[COMPLEX PARAMETERS]
0  RESIZE  =  NANC/NPOPPED  hide
1  TDIVFP  =  TDIVSF+DELTA_T  output
```

*.tpl file

```
//Number of population samples (demes)
3
//Population effective sizes (number of genes)
```

```
NPOPFOE
NPOPSCA
NPOPPED
//Sample sizes
14
12
30
//Growth rates          : negative growth implies population expansion
0
0
0
//Number of migration matrices : 0 implies no migration between demes
2
//Migration matrix 0
0 0 0
0 0 MIGSP
0 0 0
//Migration matrix 1: No migration
0 0 0
0 0 0
0 0 0
//historical event: time, source, sink, migrants, new size, new growth rate, migr. matrix
3  historical event
TDIVSF 1 0 1 1 0 1
TDIVFP 0 2 1 1 0 1
TDIVFP 2 2 1 RESIZE 0 1
//Number of independent loci [chromosome]
1 0
//Per chromosome: Number of linkage blocks
1
//per Block: data type, num loci, rec. rate and mut rate + optional parameters
FREQ 1 0 2.5e-8 OUTEXP
```

Model III. *C. x scabridifolia* as sister to *C. pedatifolia*, receiving gene flow from *C. foetidissima*

*.est file

```
// Priors and rules file
// *********************

[PARAMETERS]
//#isInt? #name   #dist.#min  #max
//all Ns are in number of haploid individuals
1  NPOPPED  unif 10  100000 output
1  NPOPSCA  unif 10  100000 output
1  NPOPFOE  unif 10  100000 output
1  NANC     unif 10  100000 output
1  TDIVSP   unif 10  100000 output
1  DELTA_T  unif 10  100000 output
0  MIGSF logunif  1e-20 1e-1 output


[RULES]

[COMPLEX PARAMETERS]
0  RESIZE  =  NANC/NPOPFOE  hide
1  TDIVPF  =  TDIVSP+DELTA_T output
```

*.tpl file

```
//Number of population samples (demes)
3
//Population effective sizes (number of genes)
NPOPFOE
NPOPSCA
NPOPPED
//Sample sizes
14
12
30
//Growth rates          : negative growth implies population expansion
0
0
0
//Number of migration matrices : 0 implies no migration between demes
2
//Migration matrix 0
0 0 0
MIGSF 0 0
0 0 0
//Migration matrix 1: No migration
0 0 0
0 0 0
0 0 0
//historical event: time, source, sink, migrants, new size, new growth rate, migr. matrix
```

```
3  historical event
TDIVSP 1 2 1 1 0 1
TDIVPF 2 0 1 1 0 1
TDIVPF 0 0 1 RESIZE 0 1
//Number of independent loci [chromosome]
1 0
//Per chromosome: Number of linkage blocks
1
//per Block: data type, num loci, rec. rate and mut rate + optional parameters
FREQ 1 0 2.5e-8 OUTEXP
```

Model IV. *C. x scabridifolia* as hybrid

*.est file

```
// Priors and rules file
// *********************

[PARAMETERS]
//#isInt? #name   #dist.#min  #max
//all Ns are in number of haploid individuals
1  NPOPPED  unif 10  100000 output
1  NPOPSCA  unif 10  100000 output
1  NPOPFOE  unif 10  100000 output
1  NANC     unif 10  100000 output
1  THYB     unif 10  100000 output
1  DELTA_T  unif 10  100000 hide
0  PROPF    unif 0  1   output bounded
0  MIGFS logunif  1e-10 1e-1 output
0  MIGFP logunif  1e-10 1e-1 output
0  MIGPS logunif  1e-10 1e-1 output
0  MIGSF logunif  1e-10 1e-1 output
0  MIGPF logunif  1e-10 1e-1 output
0  MIGSP logunif  1e-10 1e-1 output

[RULES]

[COMPLEX PARAMETERS]
0  RESIZE  =  NANC/NPOPPED  hide
1  TDIV  =  THYB+DELTA_T  output
```

*.tpl file

```
//Number of population samples (demes)
3
//Population effective sizes (number of genes)
NPOPFOE
NPOPSCA
NPOPPED
//Sample sizes
14
12
30
//Growth rates          : negative growth implies population expansion
0
0
0
//Number of migration matrices : 0 implies no migration between demes
2
//Migration matrix 0
0 MIGFS MIGFP
MIGSF 0 MIGSP
MIGPF MIGPS 0
//Migration matrix 1: No migration
0 0 0
0 0 0
0 0 0
//historical event: time, source, sink, migrants, new size, new growth rate, migr. matrix
3  historical event
THYB 1 0 PROPF 1 0 1
THYB 1 2 1 1 0 1
TDIV 0 2 1 RESIZE 0 1
//Number of independent loci [chromosome]
1 0
//Per chromosome: Number of linkage blocks
1
//per Block: data type, num loci, rec. rate and mut rate + optional parameters
FREQ 1 0 2.5e-8 OUTEXP
```

D) Compare the likelihood and AIC distributions of the observed SFS under the best parameter values of first and second-ranked models.

Most of the following code was obtained from Ravinet & Meier (2022). Speciation & Population Genomics: a how-to-guide. In: https://speciationgenomics.github.io/fastsimcoal2/ (last consulted may 18th 2023).

For each model:

Copy *.obs file with the SFS and *_maxL.par file from the run with the highest likelihood among the 50 replicates.

```
#!/bin/bash

PREFIX="BestFitModel"

# Run FastSimCoal 100 times to get the likelihood of the observed SFS under the best parameter values
# with 1 million simulated SFS.
# Use -m if using folded SFS, use -d if using unfolded SFS

for iter in {1..100}
do
fsc26 -i ${PREFIX}_maxL.par -n1000000 -m -q
 # Fastsimcoal will generate a new folder called ${model}_maxL and write files in there
 #collect the lhood values (Note that >> appends to the file, whereas > would overwrite it)
 sed -n '2,3p' ${PREFIX}_maxL/${PREFIX}_maxL.lhoods >> ${PREFIX}.lhoods
 # delete the folder with results
 rm -r ${PREFIX}_maxL/
done
```

Now you can plot the distribution of the likelihood of the SFS under the BestFitModel (*.lhoods) and SecondBestFitModel (corresponding *.lhoods).

E) Run parametric bootstrap for Confidence Interval of parameter values of BestFitModel

Create 100 simulated SFS under best parameter values.

```
#!/bin/bash


#parametric bootstrap for confidence intervals, 100 simulated SFSs under best parameters, and for each SFS
recalculate best parameters among 20 independent runs

mv BestFitModel_maxL.par BestFitModel_boot.par
fsc26 -i BestFitModel_boot.par -n 100 -m -s 254 -x -I -q -j

#FastSimCoal will generate one folder for each simulation (100 folders)
```

Retrieve *.obs files with simulated SFS and copy *.est and *.tpl files of BestFitModel

```
#!/bin/bash

#make sure original *.tpl and *.est files of BestFitModel are in same folder where boot folders are located
#If using unfolded SFS, use corresponding prefix *_DAFpop0.obs

for i in {1..100}
do
mkdir bs$i
```

```
  cd bs$i
cp ../BestFitModel_boot_${i}/BestFitModel_boot_MAFpop0.obs BestFitModel_boot${i}_MAFpop0.obs
cp ../BestFitModel.est BestFitModel_boot${i}.est
cp ../BestFitModel.tpl BestFitModel_boot${i}.tpl
cd ..
done
```

Run 20 replicates estimating parameter values for each simulated SFS

```
#!/bin/bash

for bs in {1..100}
do
  cd bs$bs
  # Run FastSimCoal 20 times:
  for i in {1..20}
  do
    mkdir run$i
    cd run$i
    cp ../BestFitModel_boot$bs*.* ./
    fsc26 -t BestFitModel_boot$bs.tpl -e BestFitModel_boot$bs.est -M -m -C 1 -n 500000 -L 70
    rm *.obs *.est *.tpl
    cd ..
  done
    cd ..
  done
```

Collect the parameter values from the best run among the 20 runs of each of the 100 replicates

```
#!/bin/bash

for bs in {1..100}
do
  cd bs$bs
  #Identify the best run with the highest MaxEstLhood.
  #In this example, MaxEstLhood is found in column no. 6 of the *.bestlhoods file.
  cat run{1..20}/BestFitModel_boot$bs/BestFitModel_boot$bs.bestlhoods | grep -v MaxEstLhood | sort -k 6 >
runsordered.txt
  sed -n 1p runsordered.txt >> ../bestrunsBestFitModel_boot.txt
  cd ..
done
```

Open the file `runsordered.txt` in text editor and add column names with parameter names accordingly (as in *.bestlhoods files).

Now you can calculate the 95% CI interval from these values. For instance, in R:

```
hist(Bestruns$NPOP)
quantile(Bestruns$NPOP, c(0.05,0.95))
```
